# Dynamic Adaptive Display System for Electrowetting Displays Based on Alternating Current and Direct Current

**DOI:** 10.3390/mi13101791

**Published:** 2022-10-20

**Authors:** Shixiao Li, Yijian Xu, Zhiyu Zhan, Pengyuan Du, Linwei Liu, Zikai Li, Huawei Wang, Pengfei Bai

**Affiliations:** Guangdong Provincial Key Laboratory of Optical Information Materials and Technology & Institute of Electronic Paper Displays, South China Academy of Advanced Optoelectronics, South China Normal University, Guangzhou 510006, China

**Keywords:** electrowetting display (EWD), alternating current (AC), direct current (DC), mixed waveform, dynamic adaptive display

## Abstract

As a representative of the new reflective display technology, electrowetting display (EWD) technology can be used as a video playback display device due to its fast response characteristics. Direct current (DC) driving brings excellent reflectivity, but static images cannot be displayed continually due to charge trapping, and it can cause afterimages when playing a dynamic video due to contact angle hysteresis. Alternating current (AC) driving brings a good dynamic video refresh ability to EWDs, but that can cause flickers. In this paper, a dynamic adaptive display model based on thin film transistor-electrowetting display (TFT-EWD) was proposed. According to the displayed image content, the TFT-EWD display driver was dynamically adjusted by AC and DC driving models. A DC hybrid driving model was suitable for static image display, which could effectively suppress oil backflow and achieve static image display while ensuring high reflectivity. A source data non-polarized model (SNPM) is an AC driving model which was suitable for dynamic video display and was proposed at the same time. Compared with DC driving, it could obtain smooth display performance with a loss of about 10 absorbance units (A.U.) of reflective luminance, which could solve the flicker problem. With the DC hybrid driving model, the ability to continuously display static images could be obtained with a loss of 2 (A.U.) of luminance. Under the AC driving in SNPM, the reflected luminance was as high as 67 A.U., which was 8 A.U. higher than the source data polarized model (SPM), and it was closer to the reflected luminance under DC driving.

## 1. Introduction

Screen display is one of the important ways for people to interact, and high-quality screen display is increasingly needed. As a representative of the new reflective display technology, electrowetting display (EWD) has high contrast ratio and response rate, which can realize the function of displaying pictures and playing videos [1,2,3]. Technologies such as liquid crystal display (LCD), organic light-emitting diode (OLED), and electrophoretic paper display (EPD) provide more convenience for information interaction [4,5]. Compared with LCD, EWD has a higher contrast ratio in strong ambient light, and it does not need to increase power consumption to adjust brightness as LCD does [6,7,8]. The reflective display technology can further replace paper reading and contribute to low-carbon environmental protection.

EWD driving waveform has always been an important part of EWDs, which can make EWDs more grayscale, have higher contrast, and better video display effect [9,10,11]. Due to the imbalance of Laplace pressure and Maxwell pressure on the three-term contact line formed by oil, polar liquid, and hydrophobic insulator, the oil backflow problem occurs in EWDs by DC driving [12]. The EWDs fail to display static pictures directly caused by the oil backflow problem [13]. DC driving can bring higher reflectivity, which can provide higher contrast in text display and picture display. Therefore, the driving of the DC signal cannot be overlooked to provide a high-contrast display effect.

Afterimage would occur by the influence of charge trapping and contact angle hysteresis, and the related problems would be solved effectively by AC driving [14,15,16]. However, the reset signal in the AC driving provided an important contribution to solving the afterimage problem, the screen could appear to flicker with the application of the reset signal, which further affected the video viewing experience [15,16,17]. Furthermore, better dynamic display during dynamic video playback was provided by AC driving, but the reflectivity and aperture ratio would be reduced, which directly led to the reduction of image contrast.

In order to make the TFT-EWD playback device have higher contrast in text and picture display and better fluency in video playback, a dynamic adaptive display model was proposed by us. The model included DC driving waveforms suitable for displaying static pictures and AC waveforms suitable for displaying dynamic video. The driving voltage waveform was adjusted by a dynamic adaptive model depending on the output display. Then, the dynamic adaptive model was applied to the self-developed TFT-EWD playback platform for evaluation. Finally, experimental tests were conducted on various playback scenarios, and it was found that the DC driving voltage hybrid model was more suitable for image and text display compared with traditional AC driving, and the proposed model has a better display effect and contrast in display dynamic video.

## 2. Principle of EWDs

**Figure 1:**
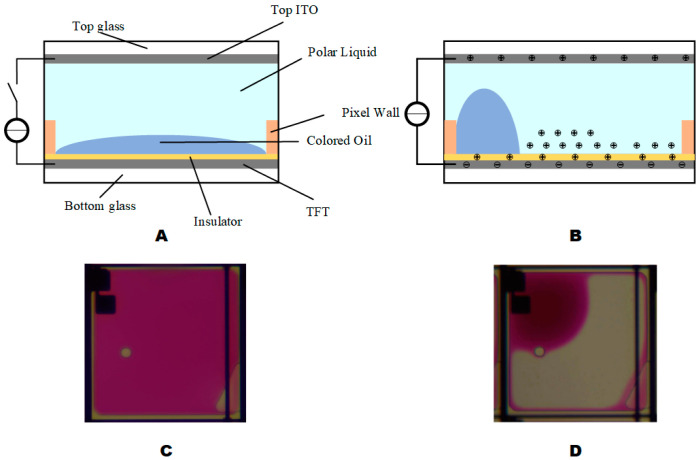
Pixel structure and operating principle of EWDs. (**A**) Pixel state when the EWD is closed. (**B**) Pixel states when the EWD is turned on. (**C**) Picture of pixel state when the EWD is closed. (**D**) Picture of pixel state when the EWD is turned on.

The electrowetting display is created by applying a driving voltage between the upper and lower ITO electrodes to change the pixel in the wettability of the polar liquid in the insulating hydrophobic layer, resulting in a change and displacement phenomenon. When voltage was applied between two electrodes of a pixel, the wettability of the polar liquid droplet can be increased. In this case, the solid–liquid interface and the dielectric layer can be taken as a parallel plate capacitor [15]. Its essence is an optical switch, which has excellent grayscale display characteristics [18]. The structure of a single pixel of EWD is shown in Figure 1A, each pixel of EWDs is primarily composed of a top plate, an indium tin oxide glass (ITO), polar liquid, colored oil, pixel wall, a hydrophobic insulator, and a lower substrate. When the voltage is not applied, the color oil within the pixel naturally covers the entire pixel and EWD will show the color of the oil, as shown in Figure 1C. When the voltage is applied, the oil moves to a pixel corner under the electric field force and the polar liquid moves to the hydrophobic layer. The contact angle between the polar liquid and the hydrophobic insulator decreases, the aperture ratio increases, and the pixel shows the color of the substrate, as shown in Figure 1D. Electrowetting is useful for making an effective display pixel [3]. Pursuing a higher aperture ratio has always been the goal of many scholars, the calculation formula for aperture ratio is shown in Equation (1) [19]:

(1) A=[1−(SoilSpix)]×100%

where A is the aperture ratio, $S_{oil}$ is the area of the oil that shrinks to the corner of the pixel, and $S_{pix}$ is the area of the pixel. Oil backflow will lead to an increase in $S_{oil}$, resulting in a decrease in the aperture ratio.

When a voltage is applied, some ions will be trapped in the insulator, as shown in Figure 1B. A local reverse electric field is formed at the interface between the dielectric and polar liquid due to the charge trapping, electrowetting force decreases due to charge trapping when a constant voltage is applied [15]. Therefore, constant voltage is not the best driver choice. The charge is trapped in the insulator by the electric field force, the electric field intensity will be reduced inside the pixel, and the increase in the driving voltage can replenish the charge in the liquid. The charge density is calculated by Equation (2) [20].

(2) $$\sigma_{L}=\frac{\epsilon_{0}\epsilon_{r}\left(V-V_{T}\right)}{d}$$

where $\sigma_{L}$ is the charge density in liquid, $\epsilon_{0}$ is the vacuum dielectric constant, $\epsilon_{r}$ is the dielectric constant of the insulating layer, V is the driving voltage, $V_{T}$ is the potential due to charge trapping in the insulator, and d is the thickness of the insulator. The charge replenishing the insulator saturates the contact angle. Charges can be removed by electrical shortcuts on metal electrodes and insulation surfaces. The electrowetting force will also increase by the increased driving voltage. The relationship between the electrowetting force and the driving voltage as shown in Equation (3) [20].

(3) $$\gamma_{LV}\left[cos\theta_{V}-cos\theta_{0}\right]=\frac{1}{2}\frac{\epsilon_{0}\epsilon_{r}}{d}{\left(V-V_{T}\right)}^{2}$$

$\gamma_{LV}$ indicates interfacial tension between polar liquids and vapor, $\theta_{V}$ is the solid and liquid contact angle when applied voltage, and $\theta_{0}$ is the solid and liquid contact angle in the initial state.

Charge trapping can be compensated by changing the polarity drive scheme [21]. Under opposite polarity conditions, different driving energies must be applied to achieve the same degree of oil shrinkage on EWD. In the EWD of the TFT structure, the polarity of the EWD pixels can be adjusted by controlling the EWD entire panel common electrode and the TFT source drive signal, achieving good grayscale display and improved image quality through switching between positive and negative polar frames [21].

## 3. Dynamic Adaptive Display System

### 3.1. Dynamic Adaptive Display Model

**Figure 2:**
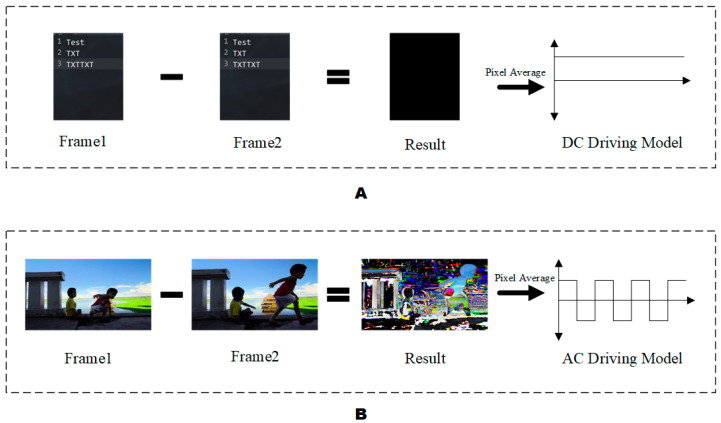
Dynamic adaptive display of the discriminant process diagram. (**A**) Diagram of the static image discrimination process. (**B**) Dynamic video discrimination process diagram.

The dynamic adaptive display method was derived from the dynamic refresh technology of LCD in mobile phones [22,23]. When displaying static text or pictures, the screen was adjusted to a lower refresh rate. When dynamic video was displayed, the LCD would provide a higher refresh rate to make the picture more vivid and smooth. The dynamic adaptive display model was judged according to the content output by the system. When displaying static text or pictures, it provided a DC driving model, which could provide better contrast. When displaying dynamic videos, an AC driving model for greater picture fluency was provided. As shown in Figure 2, Figure 2A was the discrimination process in the static image display mode, and Figure 2B was the discrimination process in the dynamic video display mode. The temporary difference method was widely used in dynamic video detection. Behavior recognition was performed by calculating the difference between the content features of the frame images before and after. Features could be analyzed by convolutional neural networks [24] or pixel subtraction [25]. Due to the consideration of the current field programmable gate array (FPGA) computing performance, this paper adopted the pixel subtraction method between frames. The calculation is shown in Equations (4) and (5).

(4) $$V_{pixel}=\frac{\sum_{0}^{h}\sum_{0}^{w}\left(\left|P_{frame1}\left(x_{i},y_{i}\right)-P_{frame2}\left(x_{i},y_{i}\right)\right|\right)}{3\times h\times w}$$

(5) $$Driving\;model=\{\begin{array}{cc} DC\;driving\;model & 0\leq V<\theta \\ AC\;driving\;model & V\geq \theta \end{array}$$

h represents the height of the image, w represents the width of the image, $P_{frame1}\left(x_{i},y_{i}\right)$ represents the pixel value of the first frame image at coordinates of $\left(x_{i},y_{i}\right)$, $P_{frame2}\left(x_{i},y_{i}\right)$ represents the pixel value of the second frame image at coordinates of $\left(x_{i},y_{i}\right),$ θ represents thresholds for judging whether the signal source is a static image, and $V_{pixel}$ stands for the average pixel difference between frames. When $V_{pixel}$ is greater than θ, the system identifies the current playback content as a dynamic video and the AC driving model is used to drive the display system. In the opposite case, the DC driving model is used.

### 3.2. DC Driving Model for Static Play

**Figure 3:**
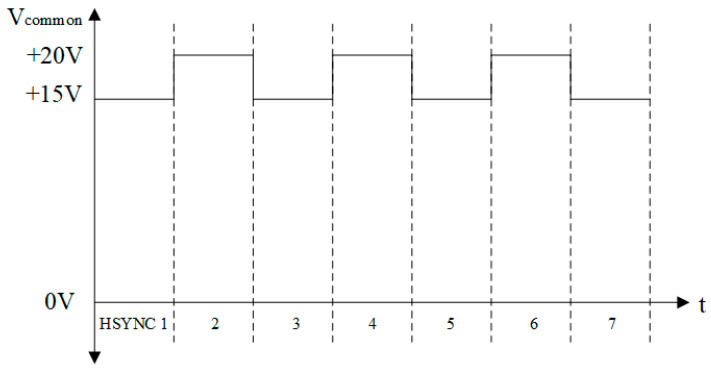
Schematic diagram of the DC hybrid driving waveform.

The reflectivity under DC driving was higher than that under AC driving, as found by researchers, and this phenomenon was also proved by experiments [9,26]. However, the problem of oil backflow under DC driving makes it impossible to maintain a static picture. Therefore, a DC-based hybrid waveform was proposed in this paper. As shown in Figure 3, based on the +15 V, a +20 V component was added. A square wave signal with a +15 V DC bias amplitude of 5 V was formed. The square wave signal can supplement the charge and prevent the occurrence of oil backflow.

### 3.3. AC Driving Models for Dynamic Displays

**Figure 4:**
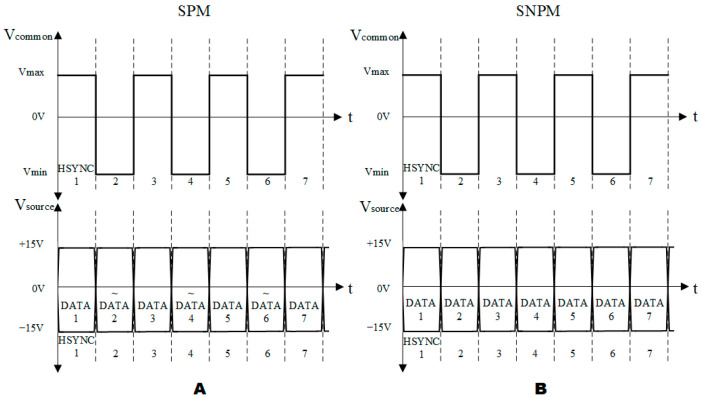
Waveform diagram of AC driving model. (**A**) Diagram of the source polarization model (SPM). (**B**) Diagram of the source non-polarized model (SNPM).

Due to the influence of contact angle hysteresis, the afterimage phenomenon would occur when playing video, which affects the playback effect of dynamic video. To cope with the occurrence of this phenomenon, an AC driving model was applied to the EWD driver [17]. The reset signal was introduced into the AC driving model, which effectively solved the problem of image sticking but would bring about the problem of video flickers. We tested the line synchronization asymmetric signal effectively to solve the problem of video afterimages and video flickers through experiments. Under the same amplitude, the aperture ratio under AC driving was lower than that of DC driving, and this phenomenon was also proved by us [27]. Therefore, we made improvements to the AC driving model. As shown in Figure 4, Figure 4A was a diagram of the source polarization model (SPM). Figure 4B was a diagram of the source non-polarized model (SNPM), the source signal did not change with the change of Vcommon. When Vcommon was switched to negative, the data in the source signal was inverted.

**Figure 5:**
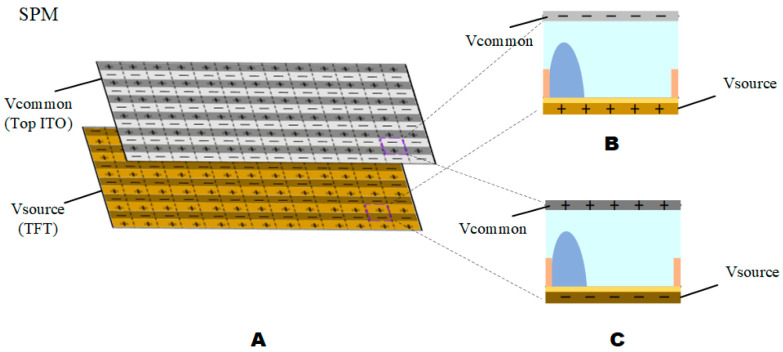
Schematic diagram of source polarization EWD model. (**A**) In TFT-EWD, Top ITO and TFT were connected to Vcommon signal and Vsource signal respectively. (**B**) When Vcommon was positive, Vsource was negative. (**C**) When Vcommon was positive, Vsource was negative.

The LCD line-by-line inversion method helps to avoid the destruction of the liquid crystal molecular characteristics [28]. This method was applied to the EWD in this paper to obtain a good display effect, as shown in Appendix A and Appendix B. As shown in Figure 5, the pixel of TFT-EWD was connected to the Vcommon and Vsource signals of top ITO and TFT, respectively. When the same content is displayed on the full screen, it was necessary to ensure that the absolute value of the voltage difference received by each pixel oil was the same. When Vcommon was the forward voltage, the source data did not need to be inverted. When Vcommon was a negative voltage, to ensure that the absolute value of the difference between the source voltage of the TFT and the common electrode voltage was the same, the source data needed to be inverted.

**Figure 6:**
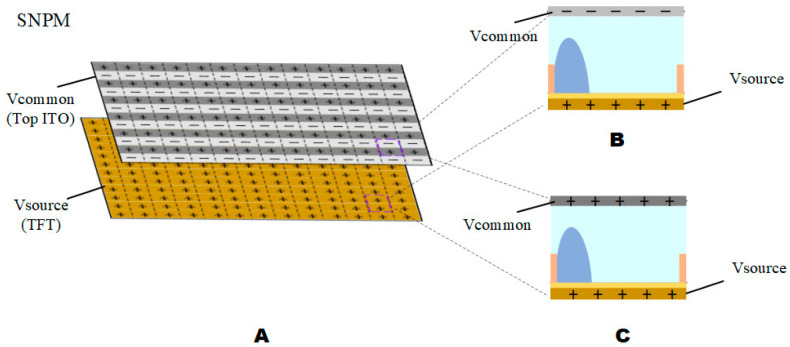
Schematic diagram of source non-polarization model. (**A**) In TFT-EWD, pixels of top ITO and TFT were connected to Vcommon signal and Vsource signal, respectively. (**B**) When Vcommon was positive, Vsource was positive. (**C**) When Vcommon was positive, Vsource was positive.

**Figure 7:**
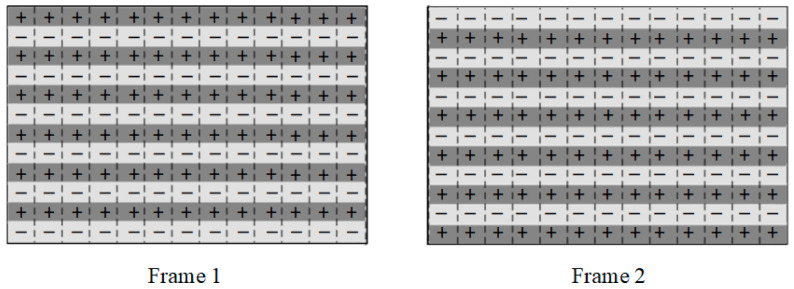
Reverse the common poles diagram of different frames.

As shown in Figure 6, the Vsource source voltage did not vary with Vcommon in SNPM. The shape of the oil changes with the absolute value of the voltage difference. It is known from the literature that oil has a millisecond response [10]. To keep the oil unchanged, the method of reversing common poles of different frames is adopted by this paper, and the unidirectional voltage of Vcommon is balanced to cause the oil shape to change. As shown in Figure 7, odd-numbered rows with positive polarity and even-numbered rows with negative polarity were adopted by the first frame, and the second frame adopts the opposite, with odd-numbered rows having negative polarity and even-numbered rows having a positive polarity. The common pole is used to quickly switch polarity to eliminate the afterimage problem caused by contact angle hysteresis.

### 3.4. Dynamic Adaptive Display Testing System

**Figure 8:**
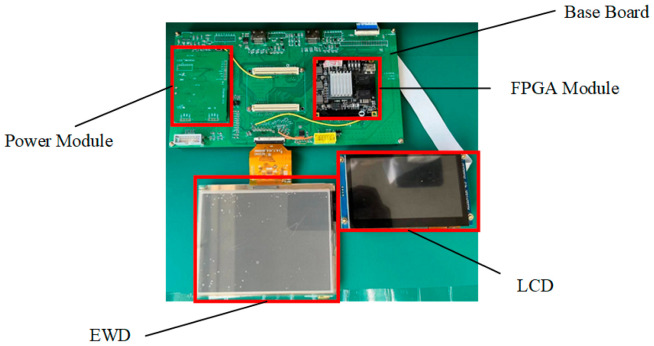
Dynamic adaptive display testing system physical map.

As shown in Figure 8, the electrowetting display system consisted of a power module, a field programmable gate array module, a substrate, an LCD, and EWDs. The power for each module was supplied by the power module. The EP4CE75F23C8 from Altera was used as a core control chip of the dynamic adaptive display testing system. The effective display resolution of EWDs was 640×480. To evaluate the effectiveness of the output signal, an LCD screen with a resolution of 800×680 was used as a signal detector to receive the same signal as EWDs.

## 4. Results and Discussion

### 4.1. DC Driving Waveform Test

**Figure 9:**
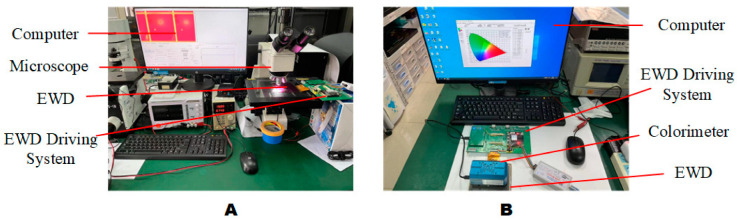
EWDs testing platform physical map. (**A**) Aperture ratio testing platform. (**B**) Reflection luminance testing platform.

In order to test the validity of the driving waveform, two testing platforms were built. As shown in Figure 9, Figure 9A was the aperture ratio testing platform, which included a computer, a microscope, EWD, and an EWD driving system. Figure 9B was the reflection luminance testing platform, which included a computer, a colorimeter, EWD, and an EWD driving system.

**Figure 10:**
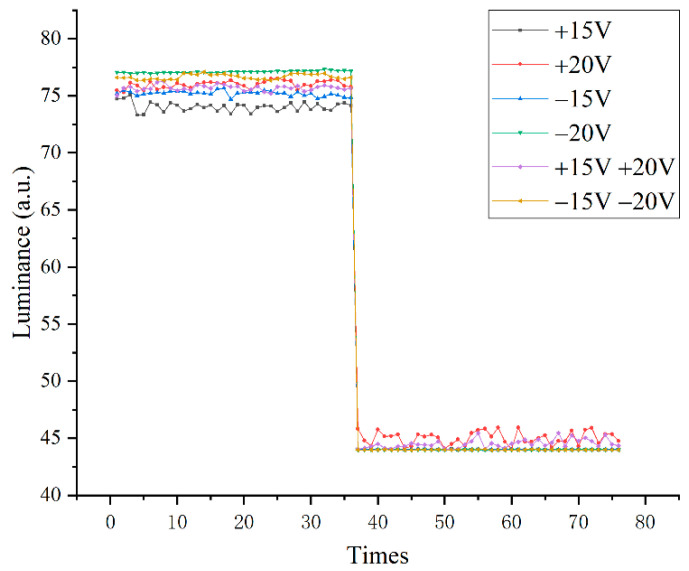
Reflectivity under each DC driving model.

EWDs were tested under DC driving and switched pixels between “on” and “off” states every second interval. Six kinds of DC driving voltages were used to drive the EWD. As can be seen from Figure 10, the luminance of the driving voltage of −20 V was the largest, followed by the combined driving voltage waveform of −15 V and −20 V, and the performance of +15 V and +20 V was relatively stable. The lowest luminance was highest for +20 V. Under the DC driving voltage, the difference in the reflected luminance of each driving waveform was not apparent.

**Figure 11:**
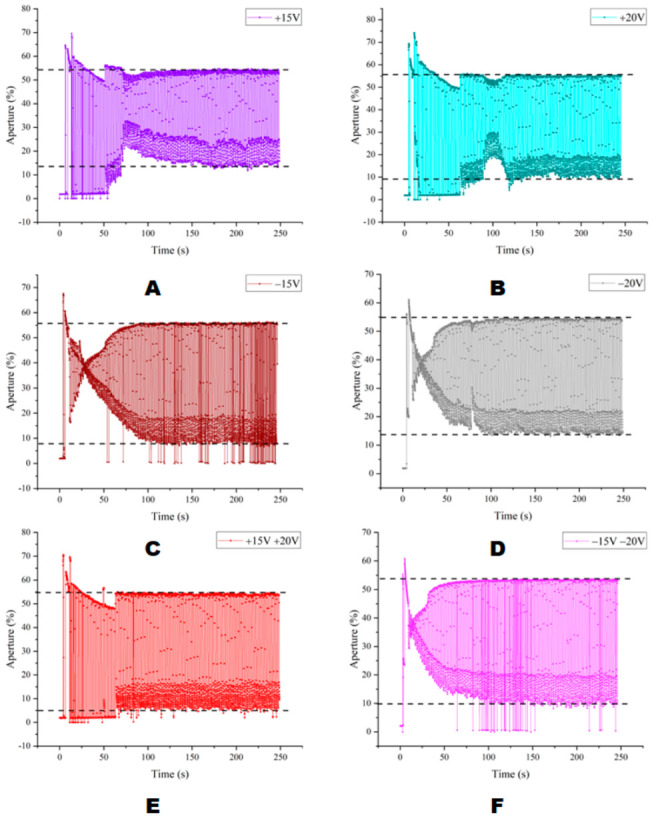
Aperture ratio in each DC driving state. (**A**) +15 V DC driving waveform. (**B**) +20 V DC driving waveform. (**C**) −15 V DC driving waveform. (**D**) −20 V DC driving waveform. (**E**) +15 V and +20 V mixed DC driving waveform. (**F**) −15 V and −20 V mixed DC driving waveform.

As shown in Figure 11, under the DC driving voltage the maximum stable aperture ratio of each driving voltage waveform could reach more than 50%. Compared with Figure 11A–D,F, the mixed waveform under the +15 V and +20 V combination represented by Figure 11E had a better consistency in the aperture ratio of the pixel “on” and “off” states. As shown in Table 1, the red data indicated that the aperture ratio data represented the best characteristics, followed by blue data. The maximum aperture ratio and average aperture ratio in EWDs on a state driven by +20 V were the best among all data, followed by +15 V and +20 V mixed waveform. However, under the +15 V and +20 V mixed waveform, the average aperture ratio in the “off” state could be as low as 8.38%. In this experiment, it was also found that the mixed waveform of +15 V and +20 V could effectively avoid the problem of oil backflow with less loss of display quality compared to +20 V.

**Figure 12:**
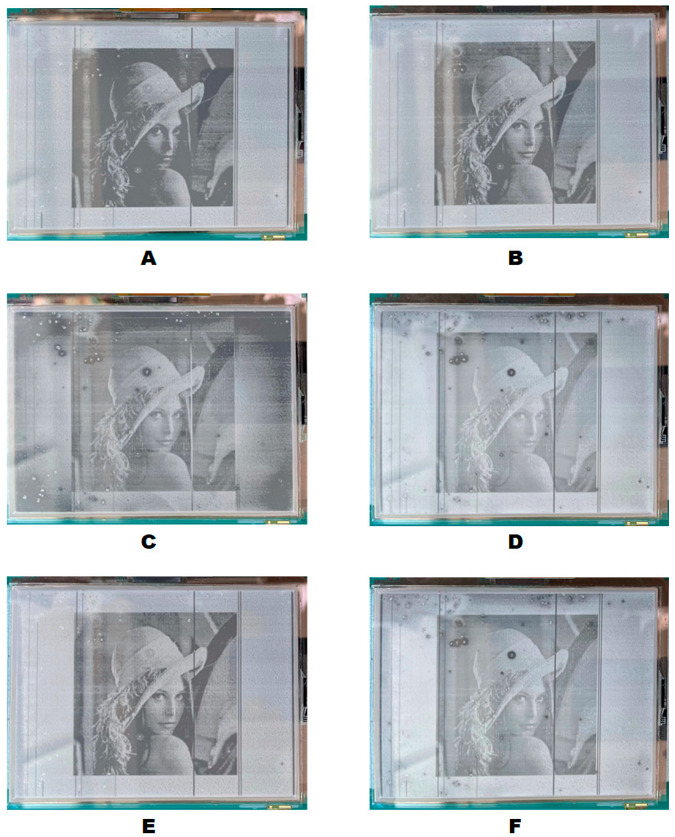
DC driving effect of static picture display. (**A**) +15 V DC driving waveform. (**B**) +20 V DC driving waveform. (**C**) −15 V DC driving waveform. (**D**) −20 V DC driving waveform. (**E**) +15 V and +20 V mixed DC driving waveform. (**F**) −15 V and −20 V mixed DC driving waveform.

As shown in Figure 12A, compared with Figure 12B,E, the image details were missing, and the overall picture was darker. Due to the obvious oil backflow phenomenon under −15 V driving conditions, the Figure 12C image was blurred. Compared to Figure 12B,E and Figure 12D,F, images had lower contrast. The image display effect of Figure 12B,E under six kinds of driving waveforms was the best. The image quality of Figure 12B,E on the visual level was basically the same, therefore, it was feasible to sacrifice a certain aperture ratio to avoid the problem of oil backflow.

### 4.2. AC Driving Waveform Test

**Figure 13:**
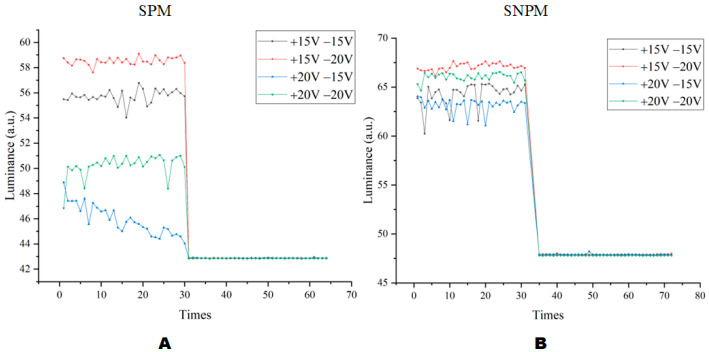
Reflected luminance graphs under various AC waveforms. (**A**) The aperture ratio of each waveform when in SPM. (**B**) The aperture ratio of each waveform when in SNPM.

It can be seen in Figure 13 that the combined waveform of +15 V and −20 V had the highest reflected luminance and the combined waveform of +20 V and −15 V had the lowest reflected luminance. When in SNPM, the reflected luminance of EWDs was higher than that in SPM. At the same time, the average reflected luminance in Figure 13B was significantly greater than that in Figure 13A under the combination of +15 V and −20 V. The average reflected luminance in Figure 13B was significantly greater than the average reflected luminance in Figure 13A under the combined waveform of +20 V and −15 V. Therefore, the SNPM could bring better-reflected luminance. In addition, it could be observed from the comparison of Figure 10 and Figure 13 that better dynamic picture display quality can be achieved at the expense of a certain amount of reflective luminance.

**Figure 14:**
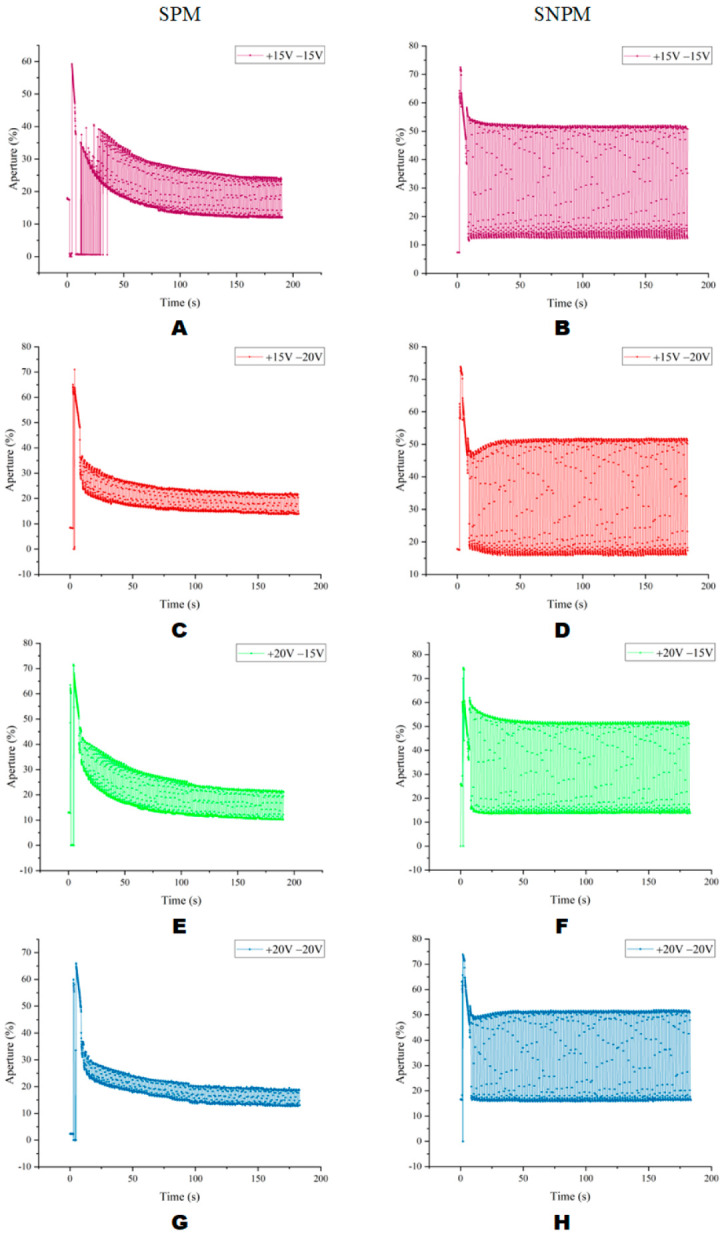
Aperture ratio under different AC driving models. (**A**) The aperture ratio of +15 V and −15 V AC driving in SPM. (**B**) The aperture ratio of +15 V and −15 V AC driving in SNPM. (**C**) The aperture ratio of +15 V and −20 V AC driving in SPM. (**D**) The aperture ratio of +15 V and −20 V AC driving in SNPM. (**E**) The aperture ratio of +20 V and −15 V AC driving in SPM. (**F**) The aperture ratio of +20 V and −15 V AC driving in SNPM. (**G**) The aperture ratio of +20 V and −20 V AC driving in SPM. (**H**) The aperture ratio of +20 V and −20 V AC driving in SNPM.

As shown in Figure 14, compared with the aperture ratio when in SPM, the aperture ratio in each AC case when in SNPM was larger. When in SNPM, the aperture ratio of the pixel in “on” and “off” states had better consistency. In addition, the difference between the aperture ratio in the “on” state and the aperture ratio in the “off” state was larger when in SNPM than in SPM, which meant there was a better response characteristic.

As shown in Table 2, in the SPM and SNPM methods, compared with other waveform combinations, the average aperture ratio of the “on” state is the highest in the case of the +20 V and −15 V combination waveform. Compared with SPM, the average aperture ratio of the “on” state under the SNPM method is 22.16% higher. Combining the results obtained in Table 2, it is possible that the best aperture ratio could be obtained in the +20 V and −15 V combined waveform.

**Figure 15:**
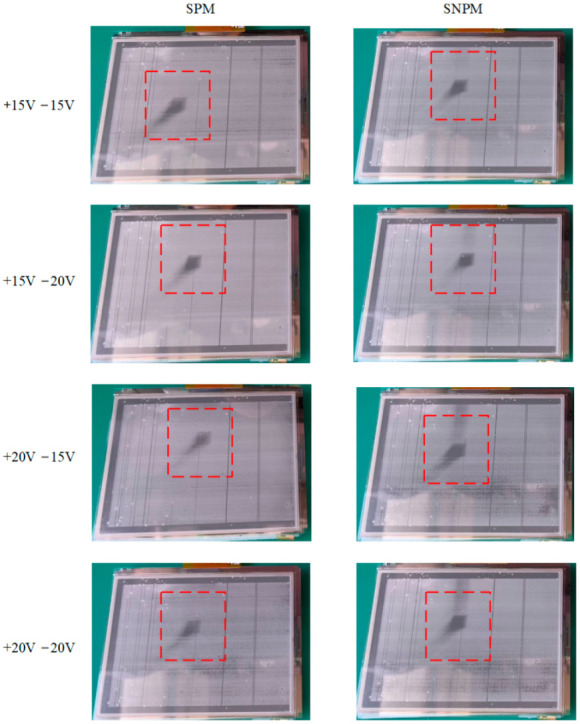
Display dynamic pictures in the AC driving models.

In this experiment, a block-moving video signal was input for the AC waveform for testing. Appendix A showed the results after using SPM with different AC driving waveforms. Appendix B showed the results after using SNPM with different AC driving waveforms. As shown in Figure 15, we used the 60-s picture as a comparison chart. In Figure 15, SPM and SNPM were used to experiment under different AC driving waveforms. Under +15 V and −15 V AC driving, compared with SPM, the boundary of the square displayed by SNPM on EWD was clearer, and the afterimage phenomenon was better suppressed. In general, the effect shown by the proposed method (SNPM) was better than that shown under SPM, but there was still an afterimage phenomenon. Under SNPM, there would be tree-shaped stripes above block graphics, which was caused by the high refresh rate.

In this AC driving test, the SNPM method causes the EWD to have better reflectivity and aperture ratio than the SPM method. In the combined waveform test, the +20 V and −15 V combined driving waveform has the best aperture ratio in both the SPM and SNPM methods, but the gap in the driving waveforms in other combinations is not obvious. In the finalization test experiment, a better display effect is obtained under the combination of +15 V and −20 V driving waveforms.

**Figure 16:**
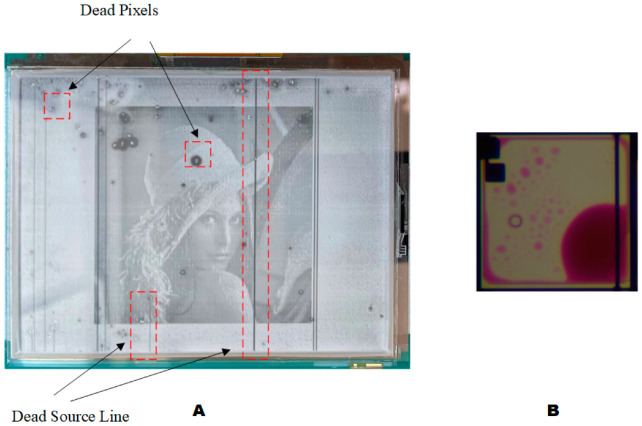
The abnormal phenomenon in the experiment. (**A**) The EWD displays anomaly analysis. (**B**) Oil splitting.

Some anomalies occurred during the experiment, as shown in Figure 16. As shown in Figure 16A, there were many dead pixels and dead source lines on the screen. The EWD preparation process and production quality were the main factors affecting the current display, resulting in the appearance of dead pixels and abnormal vertical stripes, which affect the overall appearance. Figure 16B was the phenomenon of oil splitting occurring during the aperture ratio test. Ideally, the oil shrinks in one corner of a pixel when driving a voltage is applied to EWD in the process of oil shrinkage. However, the oil may be split into two or more parts. The reason is that the charges in the hydrophobic insulator can cause a sudden change in the electric field. When the capacitance value of a pixel increases rapidly, it is likely to cause oil splitting [11].

## 5. Conclusions

In this paper, a dynamic adaptive display system for electrowetting displays based on the alternating current and the direct current was proposed. In this system, the driving model was dynamically adjusted according to the displayed content so that the EWDs had better reflection luminance when displaying a static image and better fluency when displaying a dynamic video. In addition, a hybrid DC driving model was proposed, which could effectively suppress the oil backflow, and implemented the continuous display of static images under the premise of sacrificing less reflective luminance. Finally, a source data non-polarized mode (SNPM) AC driving model was proposed, which not only solved the flicker problem when playing video but also further improved the reflected luminance of EWDs under the AC driving model.

## Figures and Tables

**Table 1:** Statistics of the aperture ratio of driving EWDs under various DC driving waveforms.

Waveforms	“On” State Maximum (%)	“Off” State Minimum (%)	“On” State Average (%)	“Off” State Average (%)
+15 V	69.5	1.82	52.76	16.14
+20 V	74.18	0	54.48	12.07
−15 V	67.61	0	51.54	14.66
−20 V	61.16	1.91	51.93	19.96
+15 V +20 V	70.55	0	53.36	8.38
−15 V −20 V	60.79	0	50.77	16.19

Red is best, blue is second best.

**Table 2:** Statistics of the aperture ratio of driving EWDs under various AC driving waveforms with different methods.

Methods	Waveforms	“On” State Maximum (%)	“Off” State Minimum (%)	“On” State Average (%)	“Off” State Average (%)
SPM	+15 V −15 V	59.23	0.56	28.69	12.78
	+15 V −20 V	70.97	0.84	26.19	16.47
	+20 V −15 V	71.58	0	29.94	14.68
	+20 V −20 V	66.02	12.64	24.37	15.71
SNPM	+15 V −15 V	72.51	11.43	52.06	13.19
	+15V −20 V	73.87	15.69	51.42	16.33
	+20 V −15 V	74.51	0	52.10	14.11
	+20 V −20 V	73.91	0	51.49	16.19

Red is best, blue is second best.

**Figure A1:**
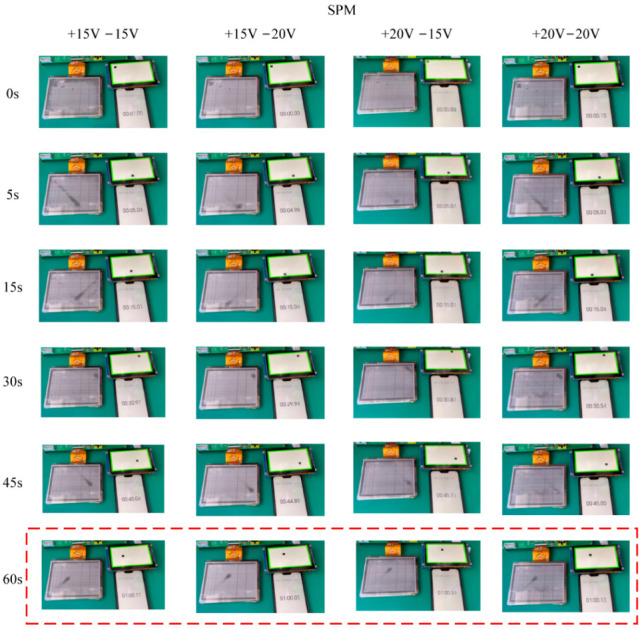
The results of the SPM applied under different AC drive waveforms.

**Figure A2:**
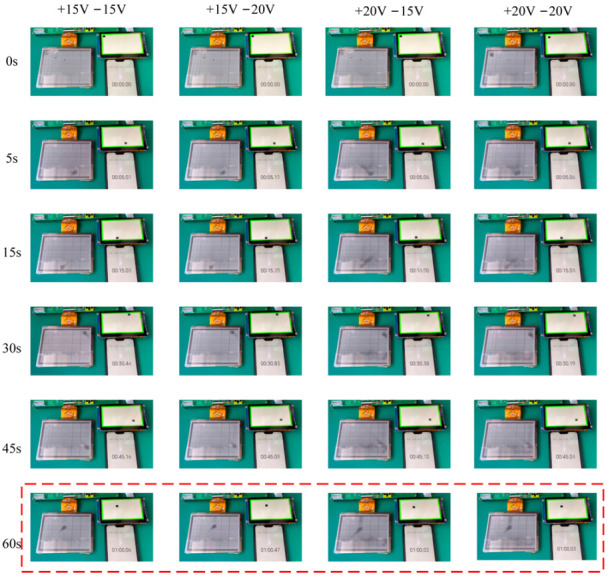
The results of applying the SNPM under different AC drive waveforms.
